# Characterization of antibody-mediated neutralization directed against the hypervariable region 1 of hepatitis C virus E2 glycoprotein

**DOI:** 10.1099/vir.0.028092-0

**Published:** 2011-03

**Authors:** Gabrielle Vieyres, Jean Dubuisson, Arvind H. Patel

**Affiliations:** 1 MRC – University of Glasgow Centre for Virus Research, University of Glasgow, Church Street, Glasgow G11 5JR, UK; 2 Institut Pasteur de Lille, Center for Infection & Immunity of Lille (CIIL), F-59019 Lille, France; 3 Inserm U1019, F-59019 Lille, France; 4 CNRS UMR8204, F-59021 Lille, France; 5 Université Lille Nord de France, F-59000 Lille, France

## Abstract

The hypervariable region 1 (HVR1) comprising the first 27 aa of E2 glycoprotein is a target for neutralizing antibodies against hepatitis C virus (HCV), but the mechanisms of this neutralization in the cell-culture-infectious genotype 2a strain JFH1 HCV virus (HCVcc) system are unknown. Two rabbit polyclonal sera, R1020 and R140, recognizing the HVR1 of the genotype 1a isolates H77c and Glasgow (Gla), respectively, and a Gla HVR1-specific mouse mAb AP213 have been described previously. However, attempts to generate of antibodies to the JFH1 HVR1 were unsuccessful. Therefore, this study produced chimeric JFH1 HCVcc viruses harbouring the H77c or Gla HVR1 to assess the reactivity of antibodies to this region and their effects on virus infectivity. The inter-genotypic HVR1 swap did not significantly affect virus infectivity. The genotype 1a HVR1-specific antibodies neutralized chimeric viruses in an isolate-dependent manner, underlining the role of HVR1 in HCV infection. The neutralizing antibodies reacted mainly with the C-terminal portion of HVR1, and detailed mapping identified A17, F20 and Q21 in the Gla HVR1 sequence and T21 (and possibly L20) in the corresponding H77c sequence as key epitope residues for AP213 and R140, and R1020, respectively. Importantly, none of the antibodies inhibited *in vitro* binding of viral envelope glycoproteins to the best-characterized HCV receptor, CD81, or to the glycosaminoglycan attachment factors. However, the HVR1 antibodies were capable of post-attachment neutralization. Overall, this study emphasizes the role of HVR1 in HCVcc entry and provides new tools to study this region further in the context of complete virions.

## INTRODUCTION

Hepatitis C virus (HCV) is a major cause of chronic hepatitis, liver cirrhosis and hepatocellular carcinoma. Genetic variability, a common feature of RNA viruses, is a major hindrance in developing effective treatments or vaccines to fight HCV. Indeed, HCV isolates are classified into seven distinct genotypes differing at the nucleotide level by around 30 % and each divided into numerous subtypes. Moreover, within a single individual, the virus exists as a constantly evolving quasispecies (Bukh *et al.*, 1995; Pawlotsky, 2003; Simmonds, 1995).

HCV, a member of the family *Flaviviridae*, has a positive-sense RNA encoding a polyprotein, which is cleaved by cellular and viral proteases into structural and non-structural proteins (reviewed by Moradpour *et al.*, 2007). The glycoproteins E1 and E2, which exhibit a high degree of variability, are responsible for virus cell attachment and entry. The retroviral HCV pseudoparticle (HCVpp) system (Bartosch *et al.*, 2003b; Hsu *et al.*, 2003) and more recently the cell-culture-infectious HCV (HCVcc) system (Lindenbach *et al.*, 2005; Wakita *et al.*, 2005; Zhong *et al.*, 2005) have shown that HCV entry into target cells is a complex process involving virus first binding to low-specificity receptors such as glycosaminoglycans (Barth *et al.*, 2003) and the low-density lipoprotein receptor (Agnello *et al.*, 1999). This is followed by sequential interaction with specific host factors such as CD81, scavenger receptor class B type I (SR-BI), claudin-1, 6 or 9, and occludin, leading to entry via the endocytic route (Evans *et al.*, 2007; Liu *et al.*, 2009; Meertens *et al.*, 2008; Pileri *et al.*, 1998; Ploss *et al.*, 2009; Scarselli *et al.*, 2002; Zheng *et al.*, 2007).

The E2 glycoprotein is the main target for the antiviral humoral immune response. Its variability is mostly confined to the so-called hypervariable regions (HVRs) (Hijikata *et al.*, 1991; Troesch *et al.*, 2006; Weiner *et al.*, 1991), which may differ by up to 80 % among HCV genotypes and even among subtypes of the same genotype. HVR1, which encompasses the first 27 aa of E2 (residues 384–410 of the viral polyprotein) contains an immunodominant epitope. Antibodies to HVR1 neutralize virus infection, but the action of this immune pressure on top of the inherent genetic instability of the virus rapidly selects resistant variants (Farci *et al.*, 2000; Kurosaki *et al.*, 1993; Rosa *et al.*, 1996; Shimizu *et al.*, 1994; Weiner *et al.*, 1992; Zibert *et al.*, 1995). Therefore, antibodies to HVR1 are typically isolate specific. In contrast, conformational epitopes on E2 are less prone to variation and are able to elicit more broadly neutralizing antibodies in patients (Johansson *et al.*, 2007; Keck *et al.*, 2007; Law *et al.*, 2008; Mancini *et al.*, 2009; Owsianka *et al.*, 2008; Perotti *et al.*, 2008; Schofield *et al.*, 2005). Interestingly, the efficiency of neutralization by antibodies targeting regions outside HVR1 is altered by interplay between HVR1, SR-BI and high-density lipoproteins (Bartosch *et al.*, 2005; Dreux *et al.*, 2006; Voisset *et al.*, 2005), emphasizing the role of HVR1 as an immunological decoy. Nevertheless, HVR1 also plays a role in HCV entry, possibly mediated by an interaction with SR-BI (Scarselli *et al.*, 2002) and/or glycosaminoglycans (Barth *et al.*, 2003). Deletion of HVR1 in HCVpp (Bartosch *et al.*, 2003c) or HCVcc (Bankwitz *et al.*, 2010) systems, or in virus produced in chimpanzee (Forns *et al.*, 2000b), results in a significant loss of infectivity. In addition, anti-HVR1 antibodies have been shown to be protective in chimpanzees (Farci *et al.*, 1996). Consistent with a role in entry, some features of HVR1, such as its overall conformation and the position of basic residues, are conserved among isolates (Callens *et al.*, 2005; Penin *et al.*, 2001).

Understanding the mechanisms of antibody-mediated neutralization is crucial to generate vaccine candidates. Whilst the mechanisms of cross-reactive neutralizing antibodies are beginning to be elucidated, the mode of action of more common isolate-specific neutralizing antibodies, mainly targeting HVR1, is not well characterized. Importantly, there are few data about anti-HVR1 antibody neutralization in the newly described HCVcc system. Therefore, in this study, the mechanism of anti-HVR1 antibody neutralization of HCVcc was investigated using a group of three antibodies in the HCVcc system, and neutralizing epitopes within HVR1 were characterized.

## RESULTS

### Generation of anti-HVR1 mAb and polyclonal sera

**Fig. 1.**
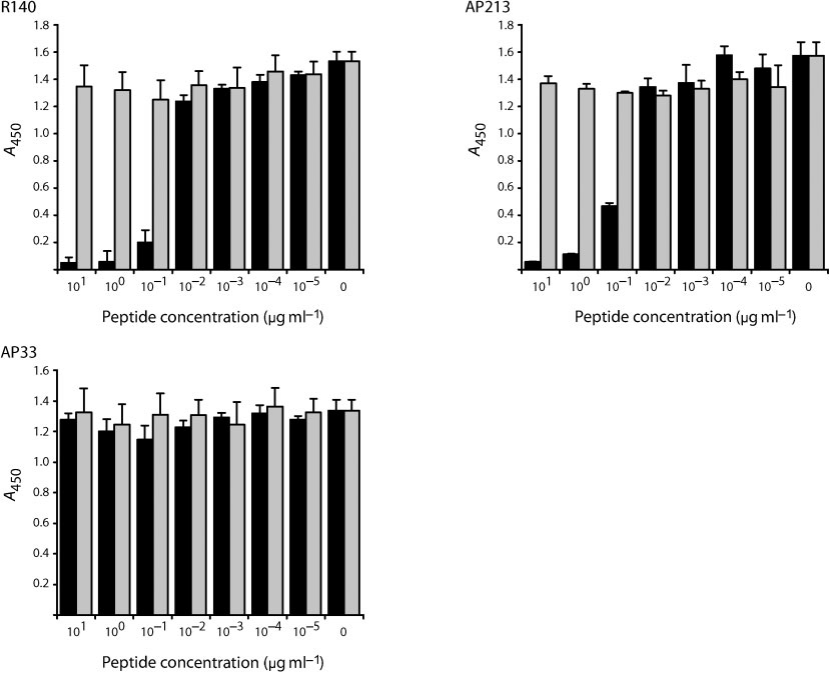
Peptide competition assay. mAbs AP33 and AP213 and the rabbit antiserum R140 were incubated with a range of concentrations of HVR1-derived (1084.B; filled bars) or irrelevant (2060.1; shaded bars) peptide. The reactivity of the peptide/antibody mixture for H77c-GlaHVR1 E1E2 (see Methods) was then tested by GNA capture ELISA.

Two polyclonal antisera, R1020 and R140, were generated by immunization of rabbits with peptides representing the HVR1 of two closely related genotype 1a strains, H77c and Glasgow (Gla), respectively. Furthermore, mAb AP213 was raised in mice immunized with a recombinant form of HCV strain Gla E1E2 (Patel *et al.*, 2000). A peptide competition assay indicated that both R140 and mAb AP213 were HVR1 specific (Fig. 1). Pre-incubation of these antibodies with peptide 1084.B representing Gla HVR1 (but not the control peptide 2060.1) prevented recognition of *Galanthus nivalis* antigen (GNA)-captured Gla E1E2 in a dose-dependent manner. As expected, neither of the peptides inhibited recognition of Gla E1E2 by mAb AP33, a broadly reactive mAb whose epitope is located immediately downstream of HVR1 (Owsianka *et al.*, 2001; Tarr *et al.*, 2006). The specificity of R1020 to strain H77c HVR1 has been demonstrated previously (Owsianka *et al.*, 2005).

### Anti-HVR1 antibodies neutralize the infectivity of chimeric HCVcc

HVR1 is a target for neutralizing antibodies in natural infections (Weiner *et al.*, 1992; Zibert *et al.*, 1995) and many anti-HVR1 antibodies are able to neutralize HCV infections *in vitro* and *in vivo*, as demonstrated in the HCVpp and chimpanzee models (Bartosch *et al.*, 2003a; Farci *et al.*, 1996; Hsu *et al.*, 2003). Despite several attempts, we were unable to raise antibodies against the strain JFH1 HCVcc HVR1. We showed previously that R1020 was able to neutralize infection of cells by HCVpp incorporating H77c E1E2 (Owsianka *et al.*, 2005). Similarly, here we found that the AP213 and R140 antibodies neutralized the infectivity of HCVpp incorporating H77c E1E2 with Gla HVR1 (see Methods and Supplementary Table S1, available in JGV Online). We set out to characterize these antibodies further using the HCVcc system.

**Fig. 2.**
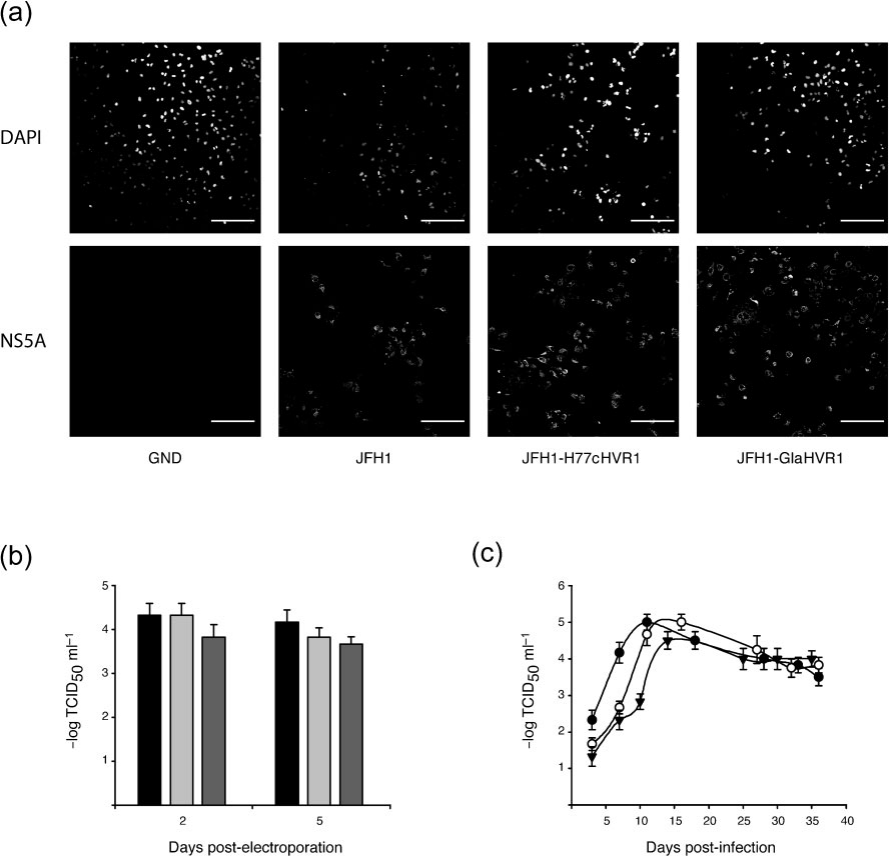
Chimeric HCVcc with genotype 1a HVR1 is infectious. (a) JFH1 chimeras were shown to replicate in Huh7 cells. Cells were fixed at 48 h after electroporation with the different constructs, stained for nuclei (DAPI; top row) and for NS5A (bottom row), and analysed by confocal microscopy. Bars, 200 μm. (b) Infectivity of the cell supernatant, assessed at 2 or 5 days after electroporation with JFH1 chimeras. Filled bars, WT JFH1; light grey bars, JFH1-H77cHVR1; dark grey bars, JFH1-GlaHVR1. (c) Production of infectious chimeric virus after infection of naïve Huh7 cells. Naïve cells infected at a low m.o.i. with supernatant from electroporated cells were serially passaged and infectious virus yields were measured in the cell supernatant at each passage by TCID_50_ assay. •, WT JFH1; ○, JFH1-H77cHVR1; ▾, JFH1-GlaHVR1.

Complete envelope proteins from the Gla strain form non-functional aggregates (Patel *et al.*, 2000). Therefore, we generated chimeric JFH1 cDNA in which its HVR1-encoding region was swapped for the corresponding H77c or Gla sequence (JFH1-H77cHVR1 or JFH1-GlaHVR1, respectively) to study the effect of the R1020 or AP213 and R140 antibodies on HCVcc infection. The viral NS5A protein was detected in Huh7 cells electroporated with RNA derived from constructs representing the wild-type (WT) JFH1 or the JFH1 HVR1 chimeras, but not from the replication-deficient GND mutant (Wakita *et al.*, 2005), indicating that the chimeras were replication competent (Fig. 2a). Moreover, after electroporation, the infectious virus titres released by both chimeras into cell culture medium at 2 or 5 days post-electroporation were comparable to those for WT JFH1 (Fig. 2b). Next, we monitored the spread of infection of the WT and chimeric viruses (released from the electroporated cells) by infecting naïve cells. Huh7 cells infected at an m.o.i. of 0.005 with different viruses were passaged over a period of 5 weeks. Measurement of the infectious virus yield in the medium at each passage (Fig. 2c) showed that the chimeric viruses spread efficiently to naïve cells, albeit with a slight delay compared with WT JFH1. Importantly, after seven passages, no mutation was found in the envelope-encoding region of these infectious chimeric viruses. Moreover, electroporated cell lysates were tested by ELISA for reactivity with anti-HVR1 antibodies. As expected, R1020 serum specifically reacted with JFH1-H77cHVR1 lysates, and R140 and AP213 antibodies specifically recognized JFH1-GlaHVR1 lysates, whilst AP33 mAb reacted with both chimeric constructs as well as with WT JFH1 E2 (data not shown).

**Fig. 3.**
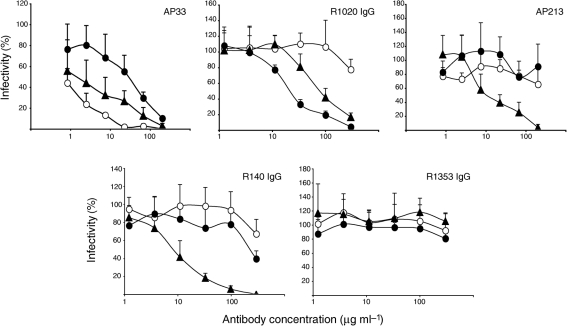
Anti-HVR1 antibodies neutralize chimeric HCVcc infection. (b) HCVcc harbouring the JFH1 (○), H77c (•) or Gla (▴) HVR1 were incubated with a range of concentrations of anti-HVR1 or control antibodies prior to infection of naïve Huh7 cells. At 2 days post-infection (p.i.), infectivity was evaluated by f.f.u. assay. The residual infectivity relative to infectivity in the absence of antibody is shown.

Therefore, these chimeric HCVcc were suitable to study the neutralizing effect of our group of anti-HVR1 antibodies. mAb AP33, known to inhibit JFH1 infection (Dhillon *et al.*, 2010; Iro *et al.*, 2009; Tarr *et al.*, 2006; Vieyres *et al.*, 2009), served as a control and was able to neutralize WT and the chimeric HCVcc (Fig. 3). However, neutralization of the chimeric HCVcc was not as potent as for WT JFH1, with at least a tenfold increase in the IC_50_ value. R1020 IgGs neutralized JFH1-H77cHVR1 virus and to a lesser extent the heterologous JFH1-GlaHVR1 virus in a dose-dependent fashion. mAb AP213 and R140 IgGs neutralized only the JFH1-GlaHVR1 virus.

### Stringent isolate specificity of anti-HVR1 antibodies

**Fig. 4.**
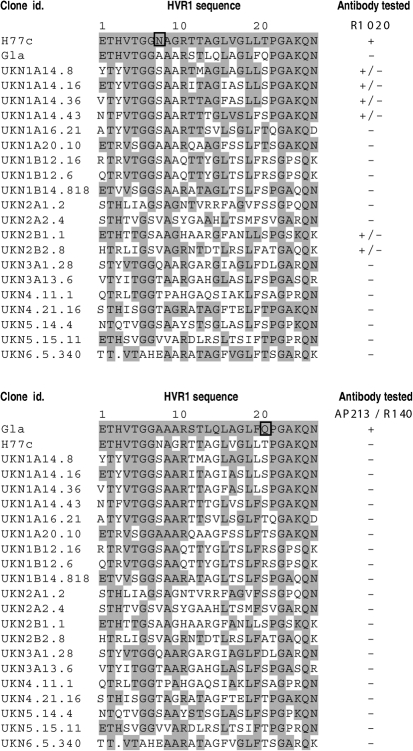
Reactivity of anti-HVR1 antibodies with a panel of patient-derived E1E2 glycoproteins. A panel of E1E2 constructs (Lavillette *et al.*, 2005; Owsianka *et al.*, 2005) was expressed in HEK cells and the cell lysates analysed by GNA ELISA for their recognition by anti-HVR1 antibodies. HVR1 sequences were aligned with the H77c (top panel) or Gla (bottom panel) HVR1 sequence and identical residues are shaded in grey. Residues specific for the H77c or Gla HVR1 are boxed. Clones were classified into three groups depending on whether they were non-reactive (–), weakly reactive (+/−) or strongly reactive (+) with anti-HVR1 antibodies in the linear range of the assay.

To gain further insights into the role of HVR1 in HCV infection, we set out to identify the amino acid residues critical for interaction with our anti-HVR1 antibodies. First, the reactivity of anti-HVR1 antibodies was assessed against a panel of 25 E1E2 constructs representing the six major genotypes of HCV, with non-redundant HVR1 sequences (Lavillette *et al.*, 2005; Owsianka *et al.*, 2005) (Fig. 4). Expression of 20 of these clones was validated by strong reaction with AP33 mAb in ELISA and these clones were therefore kept for further analysis. The AP213 and R140 antibodies reacted only with the Gla strain. Interestingly, residue Q21 was the only Gla-specific residue in this panel of HVR1 sequences and could account for the restricted isolate-specificity of the AP213 and R140 antibodies. In contrast, some cross-reactivity was observed for the R1020 antiserum, which weakly recognized some strains belonging to genotypes 1a and 2b. Interestingly, the presence of the L20 residue seemed to correlate with some epitope recognition as it was present in the H77c HVR1 as well as in four of the six clones weakly recognized by R1020 IgGs, but absent from all the negative clones. Only residue N8 was H77c specific, and this could explain the strong reactivity of the R1020 antiserum with this strain. Together, these data confirmed the isolate specificity of our anti-HVR1 antibodies.

### AP213 mAb epitope mapping by phage display

**Fig. 5.**
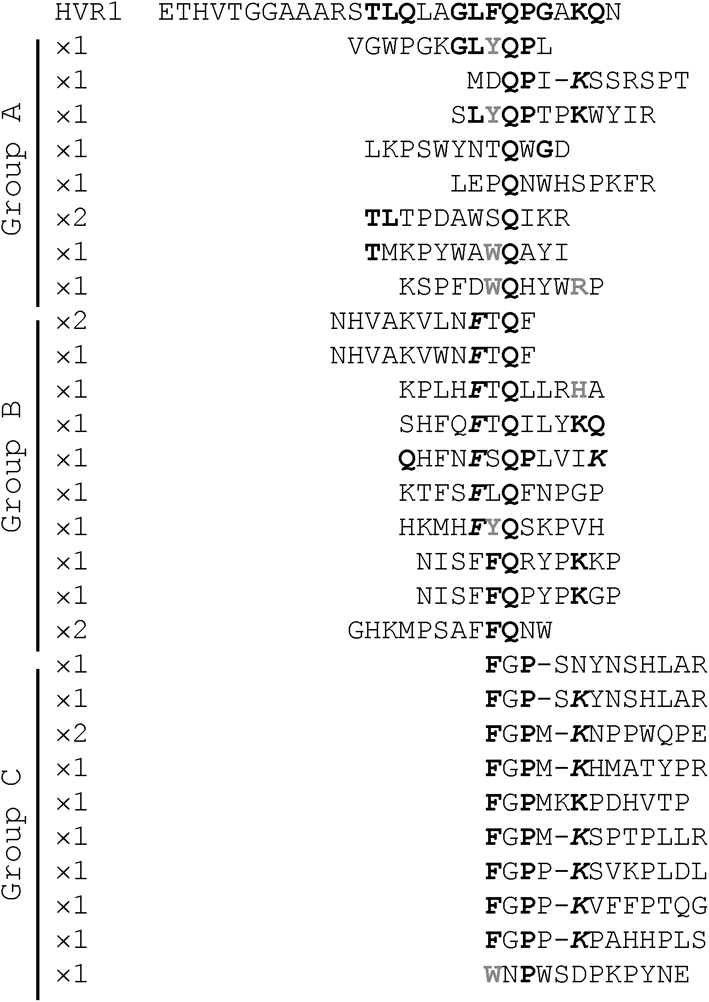
Epitope mapping of mAb AP213 by peptide phage display. A total of 32 peptides were selected after three rounds of panning of a random peptide phage display library against mAb AP213. Their amino acid sequence was aligned manually with the Gla HVR1 sequence. Residues in bold black represent residues present in the HVR1 sequence, aligned with or close to (italics) the HVR1 residue. Residues in bold grey are residues aligned with HVR1 residues having similar properties.

We next used the phage display technique to map more precisely the epitope residues recognized by our antibodies. Because R1020 and R140 are polyclonal antibodies and this method is more suitable for mAbs, we restricted our analysis to mAb AP213. Following three rounds of affinity selection against mAb AP213, 32 peptide sequences were isolated (Fig. 5). Overall, the frequency of phenylalanine (F), lysine (K) and proline (P) residues present in the HVR1 sequence was significantly increased in the selected peptides compared with the original library (at least doubled), suggesting that they were indeed selected during panning. The sequences were arranged into three groups. In groups A (nine clones) and B (12 clones), a glutamine residue (Q) was strictly conserved, often preceded by a large aromatic residue [tyrosine (Y) or tryptophan (W) in group A and phenylalanine (F) in group B] and sometimes followed by a proline residue (four sequences). Finally, group C (11 clones) exhibited an ‘FGP’ motif in all but one clone, which resembled again the ‘FQP’ motif in the Gla HVR1. We also found an overall frequent repetition of basic residues [lysine (K), arginine (R) and histidine (H)], often aligning with K25 in HVR1. Although heterogeneous, these data suggest that the ^20^FQPXXK^25^ motif in HVR1 is a key element in mAb AP213 binding.

### The C-terminal moiety of HVR1 is the main determinant for anti-HVR1 antibody binding

**Fig. 6.**
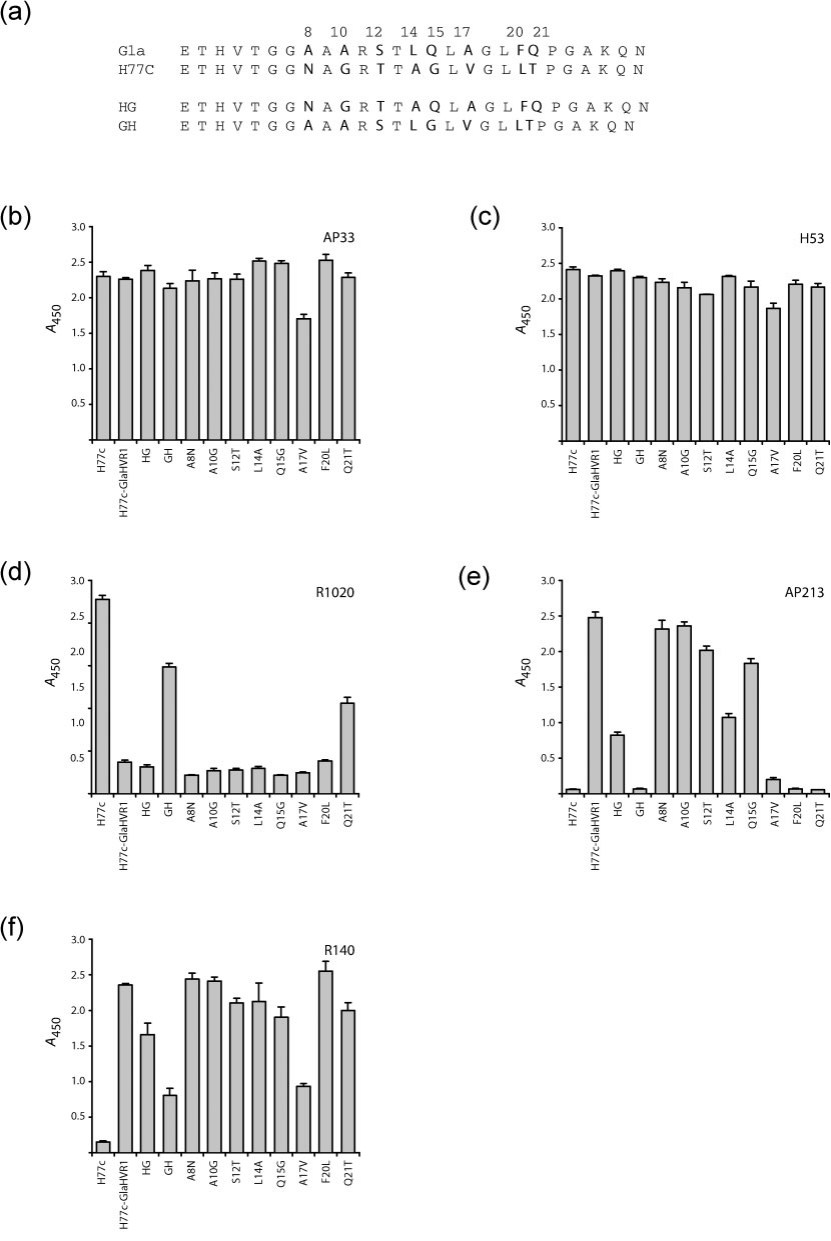
Reactivity of anti-HVR1 antibodies for a panel of E1E2 mutants. (a) Amino acid sequences of the H77c, Gla and chimeric HG or GH HVR1 (see Methods). Residues in bold differ between the H77c and Gla HVR1 sequence. All mutants were generated in the H77c-GlaHVR1 background. Clones A8N to Q21T consisted of H77c-GlaHVR1 E1E2 with single mutations in HVR1 replacing a residue from the Glasgow strain by its H77c counterpart. (b–f) Serially diluted lysates of HEK cells transfected with different E1E2 constructs were tested for recognition by different antibodies by GNA capture ELISA. For each antibody, a lysate dilution was chosen that gave absorbance readings in a linear range, and absolute absorbance values (at 450 nm) were plotted.

To delineate the epitope region of all three anti-HVR1 antibodies, a panel of E1E2 mutants was generated in a phCMV vector. They encoded, in an H77c background, a chimeric HVR1 consisting of the H77c HVR1 N terminus and the Gla HVR1 C terminus (HG chimera) or the Gla HVR1 N terminus and the H77c HVR1 C terminus (GH chimera) (Fig. 6a). Reactivity with mAb AP33 and the conformation-sensitive mAb H53 suggested that the protein expression levels and global conformation were similar to those for the parental (H77c) constructs (Fig. 6b, c). The chimeras were also functional in the HCVpp system, with at least 50 % of the infectivity of the parental strain preserved (data not shown). Nevertheless, differences in affinity for anti-HVR1 antibodies were observed. R1020 serum, specific for the H77c HVR1, did not recognize the HG chimera but reacted with the GH chimera (Fig. 6d). Importantly, residue N8, which was H77c specific (see Fig. 4 and above), was therefore not necessary for R1020 IgG binding. The Gla HVR1-specific mAb AP213 only recognized the HG chimera (Fig. 6e). Lastly, the R140 serum bound both constructs, with an increased affinity for the HG chimera (Fig. 6f). This indicated that the HVR1 C terminus was involved in antibody binding and strain specificity of all three antibodies, whilst the HVR1 N terminus was not sufficient for binding. Importantly, antibody affinity for the HVR1 chimeras was always lower than for the parental homologous complete HVR1. In addition, the spectrum of antibody neutralization towards HCVpp harbouring chimeric HVR1 (HG or GH chimeras; see Supplementary Table S1) correlated with the antibody affinity as assayed by ELISA (Fig. 6). Therefore, our anti-HVR1 antibodies reacted principally with the C-terminal moiety of HVR1, although the N-terminal portion may be important for HVR1 global conformation and optimal antibody binding affinity.

### Single point mutants in HVR1 highlight residues important for antibody binding

Each of the eight residues differing between the H77c and Gla HVR1 sequence (Fig. 6a) were individually mutated, in the H77c–Gla HVR1 sequence, to their H77c counterpart. Antibody binding to these mutants was then analysed (Fig. 6b–e). None of these mutations significantly affected the protein expression levels (mAb AP33 binding) or the affinity for the conformation-sensitive mAb H53. However, mutation of F20 and Q21 into the H77c residue resulted in a complete loss of affinity for AP213 mAb (Fig. 6e). Moreover, the A17V mutation significantly decreased the Gla HVR1 affinity for both the AP213 and R140 antibodies (Fig. 6e, f). Conversely, a single point mutation in the Gla HVR1 sequence (Q21T) could restore some affinity for R1020 antibody, which is H77c specific, indicating that residue T21 plays an important part in the R1020 epitope.

### Anti-HVR1 antibodies are partially conformation sensitive

**Fig. 7.**
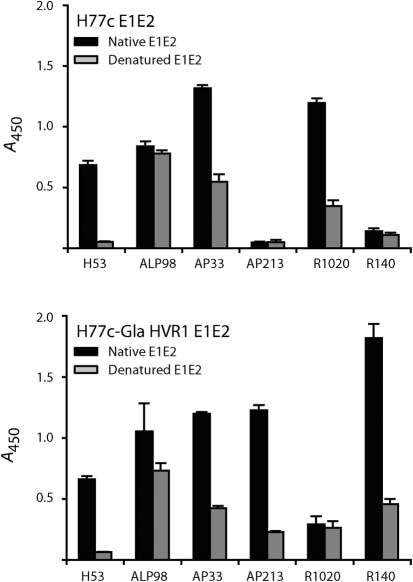
Conformation sensitivity of anti-HVR1 antibodies. Native or denatured HEK cell lysates expressing the H77c (top panel) or H77c-GlaHVR1 (bottom panel) E1E2 were captured on GNA-coated plates and tested for recognition by different antibodies. For each antibody, a lysate dilution was chosen that gave absorbance readings in a linear range and absolute absorbance values (at 450 nm) were plotted.

To test whether anti-HVR1 antibodies were conformation sensitive, their affinity for native or denatured E1E2 captured on GNA-coated plates was determined (Fig. 7). As expected (Cocquerel *et al.*, 1998), the conformation-sensitive mAb H53 specifically recognized native H77c E1E2. The affinity of mAb ALP98 for both H77c and H77c-GlaHVR1 E1E2 was only mildly affected by antigen denaturation. As shown previously (Tarr *et al.*, 2006), mAb AP33 exhibited partial conformation sensitivity for both antigens, although its epitope is linear. Interestingly, although two of them were raised against peptides, all three anti-HVR1 antibodies were also partially conformation sensitive. Of note, the peptides used to raise the R1020 and R140 antisera were relatively long (27 and 21 residues, respectively) and might fold in solution.

### Anti-HVR1 antibodies are capable of post-attachment neutralization

**Fig. 8.**
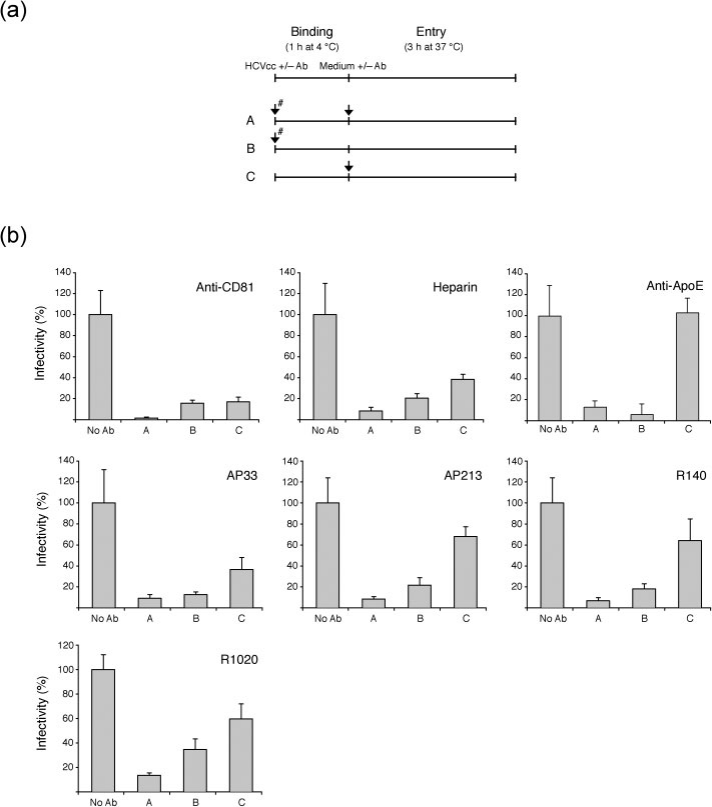
Anti-HVR1 antibodies are capable of post-attachment neutralization. (a) Schematic representation of the protocols used to distinguish between binding and entry stages of HCVcc entry (see Methods). Arrows correspond to the addition of inhibitors. The # symbol indicates that the inhibitor was pre-incubated with the appropriate HCVcc (heparin, 500 μg ml^−1^; anti-ApoE serum, 1 : 150; mAb AP33, 20 μg ml^−1^; mAb AP213, 20 μg ml^−1^; R1020 IgGs, 30 μg ml^−1^; R140 IgGs, 30 μg ml^−1^) or with the cells (anti-CD81 antibody at 5 μg ml^−1^) for 30 min at 37 °C before virus inoculation. Ab, Antibody. (b) HCVcc infectivity was assessed after adding the inhibitors at different stages of the entry process, according to protocols A, B and C. Infectivity was assessed by f.f.u. assay at 48 h p.i. and plotted as the percentage of infectivity observed in the absence of inhibitor.

To determine whether anti-HVR1 antibodies act by inhibiting HCVcc attachment or at a post-binding entry step, three protocols were used (Fig. 8, and see Methods) to distinguish between these events. Protocol C, in particular, measured post-attachment neutralization. mAb AP33 was efficient at inhibiting entry in protocol C, showing that this mAb was capable of post-attachment neutralization, as reported previously (Haberstroh *et al.*, 2008). Nevertheless, AP33-mediated neutralization was more efficient in protocol B, suggesting an additional effect of this antibody in attachment inhibition or that its epitope was more readily available before virus attachment. Interestingly, anti-HVR1 antibodies behaved similarly to mAb AP33 in that they were capable of post-attachment neutralization but were more efficient at inhibiting HCVcc entry when present during the virus-binding stage. It should be noted that the antibodies were used at suboptimal concentrations so that the differences in neutralization between protocols B and C could be observed more easily, but up to 80 % post-attachment neutralization could be obtained with anti-HVR1 antibodies used at higher concentrations (data not shown). Also, because antibodies were used at suboptimal concentrations, more efficient neutralization was observed in protocol A where antibody was added twice in the experiment. Consistent with their role in binding inhibition (Chang *et al.*, 2007; Koutsoudakis *et al.*, 2006), heparin and the anti-ApoE serum were more efficient in protocol C. Note that we did observe some post-attachment neutralization with heparin that could correspond to the elution of a fraction of the cell-bound viruses. This effect was weaker or undetected in previous reports (Haberstroh *et al.*, 2008; Koutsoudakis *et al.*, 2006; Zeisel *et al.*, 2007), possibly due to the use of different virus strains (Luc-Jc1 versus JFH-1 virus). In contrast, anti-CD81 mAb had similar effects in protocols B and C, consistent with the established role of CD81 in a post-attachment step of entry. Similar results were obtained with the HCVpp system (data not shown).

## DISCUSSION

### Anti-HVR1 antibodies neutralize chimeric HCVcc

Anti-HVR1 antibodies are generally isolate specific, and, to our knowledge, no animal-raised anti-HVR1 antibody has been obtained against the genotype 2a HCVcc JFH1 isolate. HVR1 plays a crucial role in virus entry and is a major target of the neutralizing antibody response *in vivo*. Therefore, the lack of information on anti-HVR1 antibody-mediated neutralization represents a significant gap in our understanding of the mechanics of virus entry.

In this study, we investigated the role of HVR1 in HCVcc entry and the mechanisms of antibody-mediated neutralization against this region. First, functional HCVcc was generated with heterologous genotype 1a HVR1. Whilst HVR1 deletion drastically impairs HCV infectivity (Bankwitz *et al.*, 2010; Bartosch *et al.*, 2003c; Forns *et al.*, 2000b; Wakita *et al.*, 2005), this inter-genotypic swap in HVR1 had little effect on virus infectivity, consistent with the flexibility of this segment, its relative independence from the rest of E2 (Forns *et al.*, 2000a; McCaffrey *et al.*, 2007) and its tolerance to certain mutations. These chimeras were therefore suitable tools to study the effect of a panel of anti-HVR1 antibodies on HCVcc infectivity as they were raised against genotype 1a HVR1.

The anti-HVR1 antibodies inhibited HCV infection in the HCVpp and HCVcc systems in an isolate-specific manner. Interestingly, their epitopes mapped mostly to the C-terminal part of HVR1, consistent with a HVR1 model where the neutralization determinants are located in this portion and not in the less variable N-terminal part. Indeed, for example, the rat anti-HVR1 mAbs 6/16, 7/59, 6/82 (reactive against HVR1 N-terminal part) and 9/86 (recognizing the whole HVR1) do not neutralize HCVpp infectivity (Hsu *et al.*, 2003). However, mAb 9/27, whose epitope includes the C-terminal part of HVR1 (Flint *et al.*, 2000), neutralizes HCVpp infection (Bartosch *et al.*, 2003a; Hsu *et al.*, 2003). This antibody also blocks binding of soluble E2 to SR-BI. The mAbs 2P24 and 15H4 recognize an epitope within the HVR1 C terminus (^23^GXXQ^26^ motif) and inhibit HCV serum particles binding to MOLT-4 cells (Li & Allain, 2005; Li *et al.*, 2001). In keeping with the latter, our phage display mapping data indicated that the ^20^FQPXXK^25^ motif is a key element of the mAb AP213 epitope. Lastly, statistical analyses have suggested the presence of two immunogenic domains on HVR1 (Penin *et al.*, 2001): the first region encompassing the 14 first residues of HVR1 and the second region starting from residue 17. The compiled data obtained with various anti-HVR1 antibodies suggested that the second region is the main neutralization determinant in HVR1.

Further fine mapping identified the residues A17, F20 and Q21 in the Gla HVR1 sequence and T21 (and possibly L20) in the corresponding H77c sequence as essential for the AP213 and R1020 epitopes, respectively. Residue A17 was also crucial for the reactivity of R140 to Gla HVR1. Whether these residues are involved directly in antibody–antigen interaction or whether they are essential to confer an appropriate configuration to the antigen is unknown. However, this fits with the interaction interface model whereby a small number of hydrophobic residues, which are rare in HVR1, would constitute the contact points between two proteins, this hotspot for binding energy being surrounded by a more hydrophilic region exposed to the aqueous solution (Clackson & Wells, 1995).

### Mechanisms of neutralization by anti-HVR1 antibodies

Anti-HVR1 antibodies had very little effect *in vitro* on CD81 binding and no effect on heparin binding (data not shown). This region has never been implicated in direct CD81 binding, although it was shown to modulate it (Bankwitz *et al.*, 2010; Roccasecca *et al.*, 2003). Consistently, we observed that mAb AP33 neutralization (which inhibits the E2–CD81 interaction) and also inhibition with a soluble form of CD81 (data not shown) were significantly attenuated with JFH1 HVR1 chimeras, although we could not detect any difference in mAb AP33 affinity for E1E2 extracted from infected cells (data not shown). Swapping the HVR1 loop might therefore increase the steric hindrance around the CD81-binding site, a phenomenon possibly accentuated at the surface of virus particles where glycoproteins might be more tightly packed together.

Similarly to mAb AP33, anti-HVR1 antibodies were capable of post-attachment neutralization, but were more efficient when present during the virus-binding stage. This could suggest that anti-HVR1 antibodies also inhibit virus binding or that their epitope is more available before virus attachment. Interestingly, we quantified viral RNA bound to the cell surface at 4 °C and found that attachment was not significantly affected by virus pre-incubation with anti-HVR1 antibodies (data not shown) but was strongly inhibited by heparin treatment (Vieyres *et al.*, 2009). Although one might expect an attenuated binding to SR-BI in presence of anti-HVR1 antibodies, it is likely that binding occurs mainly via virus-associated lipoproteins and is therefore not blocked by anti-HVR1 antibodies. Thus, the role of HVR1 in HCV infection is not limited to cell-surface attachment, through glycosaminoglycans binding for instance (Barth *et al.*, 2006; Basu *et al.*, 2004); on the contrary, this region seems to play an active role in entry.

In conclusion, the chimeric HCVcc constructs and anti-HVR1 antibodies described here constitute new tools to investigate further the role of HVR1 in the HCV life cycle. Antibodies targeting the HVR1 C terminus were able to neutralize HCVcc infectivity and notably inhibited a post-attachment step of entry, unravelling new roles for HVR1 in HCVcc infection.

## METHODS

### Cell culture and antibodies.

Human hepatoma Huh7 cells (Nakabayashi *et al.*, 1982) and human epithelial kidney (HEK) 293T cells (ATCC CRL-1573) were propagated as described elsewhere (Vieyres *et al.*, 2009). The mouse mAbs AP213, AP33, ALP98 and H53 and the rabbit polyclonal serum R1020 have been described previously (Clayton *et al.*, 2002; Cocquerel *et al.*, 1998; Owsianka *et al.*, 2005; Patel *et al.*, 2000). The rabbit polyclonal antiserum R140 was raised against a synthetic peptide (1084.B; see below) corresponding to the HCV genotype 1a strain Gla HVR1, as described previously (Patel *et al.*, 2000). The rabbit serum R1353 directed against an irrelevant protein was used as a control. The mouse monoclonal and sheep polyclonal anti-HCV NS5A antibodies were kind gifts from C. M. Rice and M. Harris, respectively (Lindenbach *et al.*, 2005; Macdonald *et al.*, 2003). Anti-CD81 mAb (clone JS-81) and anti-ApoE serum were obtained from BD Biosciences and Millipore, respectively. Alexa Fluor 488-conjugated donkey anti-mouse and anti-rabbit antibodies were purchased from Invitrogen. HRP-conjugated protein A or antibodies were from Sigma. Total IgGs from R1020, R140 and R1353 sera were purified using protein G affinity chromatography for use in neutralization assays.

### Plasmid constructs.

Plasmid phCMVcE1E2(H77c) encoding the full-length E1E2 of the genotype 1a strain H77c (Yanagi *et al.*, 1997) has been described previously (Owsianka *et al.*, 2005). The HVR1 (aa 384 – 410 of the viral polyprotein) in the E2 gene of this plasmid was replaced with the corresponding sequence of the genotype 1a Gla strain to form plasmid phCMVcE1E2(H77c-GlaHVR1). This plasmid also carried an A373V amino acid substitution in the H77c E1 gene. Chimeric HVR1 sequences were generated by fusion PCR and inserted into phCMVcE1E2(H77c). Point mutants in HVR1 were generated in the phCMVcE1E2(H77c-GlaHVR1) plasmid using a QuikChange II Site-directed Mutagenesis kit (Stratagene). E1E2-encoding plasmids were expressed by transfection of HEK cells and cell lysates were harvested 3 days later.

The plasmid pUC-JFH1 carries the full-length cDNA of the genotype 2a HCVcc strain JFH1 (Wakita *et al.*, 2005). The plasmid pUC-JFH1/GND is identical except that it carries the replication knockout GND mutation in the NS5B-encoding sequence (Wakita *et al.*, 2005). Chimeric JFH1 genomic cDNAs were constructed that carried the Gla or H77c HVR1 in the plasmid pUC-JFH1. All constructs were checked by restriction analysis and nucleotide sequencing of the E1E2-encoding region.

### Generation of HCVcc and infectivity assays.

HCVpp and HCVcc were generated essentially as described previously (Bartosch *et al.*, 2003b; Kato *et al.*, 2006). Briefly, linearized *in vitro*-transcribed HCV genomic RNA was electroporated into Huh7 cells. Cells were split at 2 days post-electroporation. The filtered supernatant harvested at 5 days post-electroporation was titrated and used to infect naïve cells at an m.o.i. of 0.005. These infected cells were serially passaged and the supernatant evaluated for virus infectivity at each passage. High-titre stocks were generated from infected cells at passages 5–7, titrated, stored in aliquots at −70 °C and used in neutralization experiments. In addition, 10^5^ naïve Huh7 cells were infected in six-well dishes with these virus stocks and total RNA was extracted after 3 days. HCV RNA corresponding to the E1E2-encoding region was reverse transcribed and amplified by PCR. The cDNA fragments obtained were purified on gels and sequenced to check for the appearance of adaptive mutations.

HCVcc infectivity was assessed as described previously by TCID_50_ or f.f.u. methods (Lindenbach *et al.*, 2005; Zhong *et al.*, 2005). In both cases, cells were fixed in methanol 2 days p.i. and immunostained for NS5A using mAb 9E10 or sheep anti-NS5A antiserum.

### Binding/entry assay.

Three protocols were used to determine whether anti-E2 antibodies inhibited virus attachment to the cells or acted post-binding. In protocol A, HCVcc was pre-incubated for 20 min at 37 °C with antibody, chilled briefly and then adsorbed onto pre-cooled target cells for 1 h at 4 °C. Unbound virus was removed by washing the cells twice with cold medium. Antibody was added again to the culture for 10 min at 4 °C, after which the cells were shifted to 37 °C to allow HCV entry to occur. In protocols B and C, antibody was only added reciprocally before or after HCVcc binding, respectively. Anti-CD81 mAb was pre-incubated with the cells rather than with HCVcc. The inoculum was replaced with fresh medium after 3 h at 37 °C. At 2 days p.i., cells were fixed and stained for counting of f.f.u.

### GNA capture ELISA.

Detection of E2 glycoprotein by ELISA was performed essentially as described previously (Patel *et al.*, 2000). Briefly, E1E2 glycoproteins from clarified lysates of transfected HEK cells were captured onto GNA lectin-coated ELISA plates (Immulon 2HB; Thermo Electro Corp.). The bound glycoproteins were detected with specific anti-E2 antibodies, followed by HRP-conjugated anti-species IgG and 3,3′,5,5′-tetramethylbenzidine substrate (Invitrogen). Reactions were stopped with 0.5 M H_2_SO_4_ and absorbance values were determined at 450 nm. To test for conformation sensitivity of antibody epitopes, HEK cells expressing E1E2 were lysed in PBS by freeze-thawing and sonication. Clarified cell lysates were either left untreated (native E1E2) or boiled for 10 min in the presence of SDS (0.1 % final) and DTT (20 mM final) (denatured E1E2). NP-40 was added to both native and denatured samples at a final concentration of 1 %. The glycoproteins were then captured on GNA-coated plates and tested for antibody recognition as described above.

### Peptide competition assay.

Branched peptides 1084.B [residues 390–410 of HCV strain Gla HVR1: (GAAARSTLQLAGLFQPGAKQN)_4_K_3_A] and 2060.10 [residues 604–621 of HCV strain H77c E2: (TPRCMVDYPYRLWHYPCT)_4_K_3_A] were incubated in a range of concentrations with mAbs AP33 and AP213 or R140 antiserum at room temperature for 30 min. The peptide/antibody mix was then tested for reactivity against GNA-captured H77c-GlaHVR1 E1E2 in an ELISA as described above.

### AP213 epitope mapping by random peptide phage display.

A PhD-12 Phage Display Peptide Library kit (New England Biolabs) was used according to the manufacturer's instructions. Briefly, the peptide library was enriched for mAb AP213-specific peptides by three rounds of panning. At each round, phages were incubated in a well of an Immulon 2HB plate (Thermo Electro Corp.) coated with 10 μg mAb AP213 ml^−1^ and bound phages were eluted in low-pH buffer. After the third round of panning, 52 eluted phages were isolated, amplified and tested by ELISA for their reactivity with mAb AP213 or an isotype control (mAb AP33). The DNA sequence of 32 AP213-specific phages was determined following the manufacturer's instructions. The deduced peptide sequences were aligned with that of Gla E1E2 using clustal w and the alignment with Gla HVR1 was further adjusted manually.

## Supplementary Material

[Supplementary Table]

## References

[r1] Agnello, V., Abel, G., Elfahal, M., Knight, G. B. & Zhang, Q. X. (1999). Hepatitis C virus and other *Flaviviridae* viruses enter cells via low density lipoprotein receptor. Proc Natl Acad Sci U S A 96, 12766–12771. 10535997 10.1073/pnas.96.22.12766 PMC23090

[r2] Bankwitz, D., Steinmann, E., Bitzegeio, J., Ciesek, S., Friesland, M., Herrmann, E., Zeisel, M. B., Baumert, T. F., Keck, Z. Y. & other authors (2010). Hepatitis C virus hypervariable region 1 modulates receptor interactions, conceals the CD81 binding site, and protects conserved neutralizing epitopes. J Virol 84, 5751–5763. 20357091 10.1128/JVI.02200-09 PMC2876602

[r3] Barth, H., Schafer, C., Adah, M. I., Zhang, F., Linhardt, R. J., Toyoda, H., Kinoshita-Toyoda, A., Toida, T., Van Kuppevelt, T. H. & other authors (2003). Cellular binding of hepatitis C virus envelope glycoprotein E2 requires cell surface heparan sulfate. J Biol Chem 278, 41003–41012. 12867431 10.1074/jbc.M302267200

[r4] Barth, H., Schnober, E. K., Zhang, F., Linhardt, R. J., Depla, E., Boson, B., Cosset, F. L., Patel, A. H., Blum, H. E. & Baumert, T. F. (2006). Viral and cellular determinants of the hepatitis C virus envelope–heparan sulfate interaction. J Virol 80, 10579–10590. 16928753 10.1128/JVI.00941-06 PMC1641783

[r5] Bartosch, B., Bukh, J., Meunier, J. C., Granier, C., Engle, R. E., Blackwelder, W. C., Emerson, S. U., Cosset, F. L. & Purcell, R. H. (2003a). In vitro assay for neutralizing antibody to hepatitis C virus: evidence for broadly conserved neutralization epitopes. Proc Natl Acad Sci U S A 100, 14199–14204. 14617769 10.1073/pnas.2335981100 PMC283569

[r6] Bartosch, B., Dubuisson, J. & Cosset, F. L. (2003b). Infectious hepatitis C virus pseudo-particles containing functional E1–E2 envelope protein complexes. J Exp Med 197, 633–642. 12615904 10.1084/jem.20021756 PMC2193821

[r7] Bartosch, B., Vitelli, A., Granier, C., Goujon, C., Dubuisson, J., Pascale, S., Scarselli, E., Cortese, R., Nicosia, A. & Cosset, F. L. (2003c). Cell entry of hepatitis C virus requires a set of co-receptors that include the CD81 tetraspanin and the SR-B1 scavenger receptor. J Biol Chem 278, 41624–41630. 12913001 10.1074/jbc.M305289200

[r8] Bartosch, B., Verney, G., Dreux, M., Donot, P., Morice, Y., Penin, F., Pawlotsky, J. M., Lavillette, D. & Cosset, F. L. (2005). An interplay between hypervariable region 1 of the hepatitis C virus E2 glycoprotein, the scavenger receptor BI, and high-density lipoprotein promotes both enhancement of infection and protection against neutralizing antibodies. J Virol 79, 8217–8229. 15956567 10.1128/JVI.79.13.8217-8229.2005 PMC1143705

[r9] Basu, A., Beyene, A., Meyer, K. & Ray, R. (2004). The hypervariable region 1 of the E2 glycoprotein of hepatitis C virus binds to glycosaminoglycans, but this binding does not lead to infection in a pseudotype system. J Virol 78, 4478–4486. 15078928 10.1128/JVI.78.9.4478-4486.2004 PMC387685

[r10] Bukh, J., Miller, R. H. & Purcell, R. H. (1995). Genetic heterogeneity of hepatitis C virus: quasispecies and genotypes. Semin Liver Dis 15, 41–63. 7597443 10.1055/s-2007-1007262

[r11] Callens, N., Ciczora, Y., Bartosch, B., Vu-Dac, N., Cosset, F. L., Pawlotsky, J. M., Penin, F. & Dubuisson, J. (2005). Basic residues in hypervariable region 1 of hepatitis C virus envelope glycoprotein E2 contribute to virus entry. J Virol 79, 15331–15341. 16306604 10.1128/JVI.79.24.15331-15341.2005 PMC1316016

[r12] Chang, K. S., Jiang, J., Cai, Z. & Luo, G. (2007). Human apolipoprotein E is required for infectivity and production of hepatitis C virus in cell culture. J Virol 81, 13783–13793. 17913825 10.1128/JVI.01091-07 PMC2168882

[r13] Clackson, T. & Wells, J. A. (1995). A hot spot of binding energy in a hormone–receptor interface. Science 267, 383–386. 7529940 10.1126/science.7529940

[r14] Clayton, R. F., Owsianka, A., Aitken, J., Graham, S., Bhella, D. & Patel, A. H. (2002). Analysis of antigenicity and topology of E2 glycoprotein present on recombinant hepatitis C virus-like particles. J Virol 76, 7672–7682. 12097581 10.1128/JVI.76.15.7672-7682.2002 PMC136371

[r15] Cocquerel, L., Meunier, J. C., Pillez, A., Wychowski, C. & Dubuisson, J. (1998). A retention signal necessary and sufficient for endoplasmic reticulum localization maps to the transmembrane domain of hepatitis C virus glycoprotein E2. J Virol 72, 2183–2191. 9499075 10.1128/jvi.72.3.2183-2191.1998 PMC109514

[r16] Dhillon, S., Witteveldt, J., Gatherer, D., Owsianka, A. M., Zeisel, M. B., Zahid, M. N., Rychlowska, M., Foung, S. K., Baumert, T. F. & other authors (2010). Mutations within a conserved region of the hepatitis C virus E2 glycoprotein that influence virus–receptor interactions and sensitivity to neutralizing antibodies. J Virol 84, 5494–5507. 20237087 10.1128/JVI.02153-09 PMC2876616

[r17] Dreux, M., Pietschmann, T., Granier, C., Voisset, C., Ricard-Blum, S., Mangeot, P. E., Keck, Z., Foung, S., Vu-Dac, N. & other authors (2006). High density lipoprotein inhibits hepatitis C virus-neutralizing antibodies by stimulating cell entry via activation of the scavenger receptor BI. J Biol Chem 281, 18285–18295. 16675450 10.1074/jbc.M602706200

[r18] Evans, M. J., von Hahn, T., Tscherne, D. M., Syder, A. J., Panis, M., Wolk, B., Hatziioannou, T., McKeating, J. A., Bieniasz, P. D. & Rice, C. M. (2007). Claudin-1 is a hepatitis C virus co-receptor required for a late step in entry. Nature 446, 801–805. 17325668 10.1038/nature05654

[r19] Farci, P., Shimoda, A., Wong, D., Cabezon, T., De Gioannis, D., Strazzera, A., Shimizu, Y., Shapiro, M., Alter, H. J. & Purcell, R. H. (1996). Prevention of hepatitis C virus infection in chimpanzees by hyperimmune serum against the hypervariable region 1 of the envelope 2 protein. Proc Natl Acad Sci U S A 93, 15394–15399. 8986822 10.1073/pnas.93.26.15394 PMC26415

[r20] Farci, P., Shimoda, A., Coiana, A., Diaz, G., Peddis, G., Melpolder, J. C., Strazzera, A., Chien, D. Y., Munoz, S. J. & other authors (2000). The outcome of acute hepatitis C predicted by the evolution of the viral quasispecies. Science 288, 339–344. 10764648 10.1126/science.288.5464.339

[r21] Flint, M., Dubuisson, J., Maidens, C., Harrop, R., Guile, G. R., Borrow, P. & McKeating, J. A. (2000). Functional characterization of intracellular and secreted forms of a truncated hepatitis C virus E2 glycoprotein. J Virol 74, 702–709. 10623732 10.1128/jvi.74.2.702-709.2000 PMC111590

[r22] Forns, X., Allander, T., Rohwer-Nutter, P. & Bukh, J. (2000a). Characterization of modified hepatitis C virus E2 proteins expressed on the cell surface. Virology 274, 75–85. 10936090 10.1006/viro.2000.0419

[r23] Forns, X., Thimme, R., Govindarajan, S., Emerson, S. U., Purcell, R. H., Chisari, F. V. & Bukh, J. (2000b). Hepatitis C virus lacking the hypervariable region 1 of the second envelope protein is infectious and causes acute resolving or persistent infection in chimpanzees. Proc Natl Acad Sci U S A 97, 13318–13323. 11078521 10.1073/pnas.230453597 PMC27222

[r24] Haberstroh, A., Schnober, E. K., Zeisel, M. B., Carolla, P., Barth, H., Blum, H. E., Cosset, F. L., Koutsoudakis, G., Bartenschlager, R. & other authors (2008). Neutralizing host responses in hepatitis C virus infection target viral entry at postbinding steps and membrane fusion. Gastroenterology 135, 1719–1728. 18718838 10.1053/j.gastro.2008.07.018

[r25] Hijikata, M., Kato, N., Ootsuyama, Y., Nakagawa, M., Ohkoshi, S. & Shimotohno, K. (1991). Hypervariable regions in the putative glycoprotein of hepatitis C virus. Biochem Biophys Res Commun 175, 220–228. 1847805 10.1016/s0006-291x(05)81223-9

[r26] Hsu, M., Zhang, J., Flint, M., Logvinoff, C., Cheng-Mayer, C., Rice, C. M. & McKeating, J. A. (2003). Hepatitis C virus glycoproteins mediate pH-dependent cell entry of pseudotyped retroviral particles. Proc Natl Acad Sci U S A 100, 7271–7276. 12761383 10.1073/pnas.0832180100 PMC165865

[r27] Iro, M., Witteveldt, J., Angus, A. G., Woerz, I., Kaul, A., Bartenschlager, R. & Patel, A. H. (2009). A reporter cell line for rapid and sensitive evaluation of hepatitis C virus infectivity and replication. Antiviral Res 83, 148–155. 19397930 10.1016/j.antiviral.2009.04.007

[r28] Johansson, D. X., Voisset, C., Tarr, A. W., Aung, M., Ball, J. K., Dubuisson, J. & Persson, M. A. (2007). Human combinatorial libraries yield rare antibodies that broadly neutralize hepatitis C virus. Proc Natl Acad Sci U S A 104, 16269–16274. 17911260 10.1073/pnas.0705522104 PMC2042196

[r29] Kato, T., Date, T., Murayama, A., Morikawa, K., Akazawa, D. & Wakita, T. (2006). Cell culture and infection system for hepatitis C virus. Nat Protoc 1, 2334–2339. 17406476 10.1038/nprot.2006.395

[r30] Keck, Z. Y., Xia, J., Cai, Z., Li, T. K., Owsianka, A. M., Patel, A. H., Luo, G. & Foung, S. K. (2007). Immunogenic and functional organization of hepatitis C virus (HCV) glycoprotein E2 on infectious HCV virions. J Virol 81, 1043–1047. 17079294 10.1128/JVI.01710-06 PMC1797433

[r31] Koutsoudakis, G., Kaul, A., Steinmann, E., Kallis, S., Lohmann, V., Pietschmann, T. & Bartenschlager, R. (2006). Characterization of the early steps of hepatitis C virus infection by using luciferase reporter viruses. J Virol 80, 5308–5320. 16699011 10.1128/JVI.02460-05 PMC1472176

[r32] Kurosaki, M., Enomoto, N., Marumo, F. & Sato, C. (1993). Rapid sequence variation of the hypervariable region of hepatitis C virus during the course of chronic infection. Hepatology 18, 1293–1299. 8244252

[r33] Lavillette, D., Tarr, A. W., Voisset, C., Donot, P., Bartosch, B., Bain, C., Patel, A. H., Dubuisson, J., Ball, J. K. & Cosset, F. L. (2005). Characterization of host-range and cell entry properties of the major genotypes and subtypes of hepatitis C virus. Hepatology 41, 265–274. 15660396 10.1002/hep.20542

[r34] Law, M., Maruyama, T., Lewis, J., Giang, E., Tarr, A. W., Stamataki, Z., Gastaminza, P., Chisari, F. V., Jones, I. M. & other authors (2008). Broadly neutralizing antibodies protect against hepatitis C virus quasispecies challenge. Nat Med 14, 25–27. 18064037 10.1038/nm1698

[r35] Li, C. & Allain, J. P. (2005). Chimeric monoclonal antibodies to hypervariable region 1 of hepatitis C virus. J Gen Virol 86, 1709–1716. 15914849 10.1099/vir.0.80912-0

[r36] Li, C., Candotti, D. & Allain, J. P. (2001). Production and characterization of monoclonal antibodies specific for a conserved epitope within hepatitis C virus hypervariable region 1. J Virol 75, 12412–12420. 11711631 10.1128/JVI.75.24.12412-12420.2001 PMC116137

[r37] Lindenbach, B. D., Evans, M. J., Syder, A. J., Wolk, B., Tellinghuisen, T. L., Liu, C. C., Maruyama, T., Hynes, R. O., Burton, D. R. & other authors (2005). Complete replication of hepatitis C virus in cell culture. Science 309, 623–626. 15947137 10.1126/science.1114016

[r38] Liu, S., Yang, W., Shen, L., Turner, J. R., Coyne, C. B. & Wang, T. (2009). Tight junction proteins claudin-1 and occludin control hepatitis C virus entry and are downregulated during infection to prevent superinfection. J Virol 83, 2011–2014. 19052094 10.1128/JVI.01888-08 PMC2643775

[r39] Macdonald, A., Crowder, K., Street, A., McCormick, C., Saksela, K. & Harris, M. (2003). The hepatitis C virus non-structural NS5A protein inhibits activating protein-1 function by perturbing ras–ERK pathway signaling. J Biol Chem 278, 17775–17784. 12621033 10.1074/jbc.M210900200

[r40] Mancini, N., Diotti, R. A., Perotti, M., Sautto, G., Clementi, N., Nitti, G., Patel, A. H., Ball, J. K., Clementi, M. & Burioni, R. (2009). Hepatitis C virus (HCV) infection may elicit neutralizing antibodies targeting epitopes conserved in all viral genotypes. PLoS ONE 4, e8254. 20011511 10.1371/journal.pone.0008254 PMC2785886

[r41] McCaffrey, K., Boo, I., Poumbourios, P. & Drummer, H. E. (2007). Expression and characterization of a minimal hepatitis C virus glycoprotein E2 core domain that retains CD81 binding. J Virol 81, 9584–9590. 17581991 10.1128/JVI.02782-06 PMC1951388

[r42] Meertens, L., Bertaux, C., Cukierman, L., Cormier, E., Lavillette, D., Cosset, F. L. & Dragic, T. (2008). The tight junction proteins claudin-1, -6, and -9 are entry cofactors for hepatitis C virus. J Virol 82, 3555–3560. 18234789 10.1128/JVI.01977-07 PMC2268462

[r43] Moradpour, D., Penin, F. & Rice, C. M. (2007). Replication of hepatitis C virus. Nat Rev Microbiol 5, 453–463. 17487147 10.1038/nrmicro1645

[r44] Nakabayashi, H., Taketa, K., Miyano, K., Yamane, T. & Sato, J. (1982). Growth of human hepatoma cells lines with differentiated functions in chemically defined medium. Cancer Res 42, 3858–3863. 6286115

[r45] Owsianka, A., Clayton, R. F., Loomis-Price, L. D., McKeating, J. A. & Patel, A. H. (2001). Functional analysis of hepatitis C virus E2 glycoproteins and virus-like particles reveals structural dissimilarities between different forms of E2. J Gen Virol 82, 1877–1883. 11457993 10.1099/0022-1317-82-8-1877

[r46] Owsianka, A., Tarr, A. W., Juttla, V. S., Lavillette, D., Bartosch, B., Cosset, F. L., Ball, J. K. & Patel, A. H. (2005). Monoclonal antibody AP33 defines a broadly neutralizing epitope on the hepatitis C virus E2 envelope glycoprotein. J Virol 79, 11095–11104. 16103160 10.1128/JVI.79.17.11095-11104.2005 PMC1193588

[r47] Owsianka, A. M., Tarr, A. W., Keck, Z. Y., Li, T. K., Witteveldt, J., Adair, R., Foung, S. K., Ball, J. K. & Patel, A. H. (2008). Broadly neutralizing human monoclonal antibodies to the hepatitis C virus E2 glycoprotein. J Gen Virol 89, 653–659. 18272755 10.1099/vir.0.83386-0 PMC2885755

[r48] Patel, A. H., Wood, J., Penin, F., Dubuisson, J. & McKeating, J. A. (2000). Construction and characterization of chimeric hepatitis C virus E2 glycoproteins: analysis of regions critical for glycoprotein aggregation and CD81 binding. J Gen Virol 81, 2873–2883. 11086118 10.1099/0022-1317-81-12-2873

[r49] Pawlotsky, J. M. (2003). Hepatitis C virus genetic variability: pathogenic and clinical implications. Clin Liver Dis 7, 45–66. 12691458 10.1016/s1089-3261(02)00065-x

[r50] Penin, F., Combet, C., Germanidis, G., Frainais, P. O., Deleage, G. & Pawlotsky, J. M. (2001). Conservation of the conformation and positive charges of hepatitis C virus E2 envelope glycoprotein hypervariable region 1 points to a role in cell attachment. J Virol 75, 5703–5710. 11356980 10.1128/JVI.75.12.5703-5710.2001 PMC114285

[r51] Perotti, M., Mancini, N., Diotti, R. A., Tarr, A. W., Ball, J. K., Owsianka, A., Adair, R., Patel, A. H., Clementi, M. & Burioni, R. (2008). Identification of a broadly cross-reacting and neutralizing human monoclonal antibody directed against the hepatitis C virus E2 protein. J Virol 82, 1047–1052. 17989176 10.1128/JVI.01986-07 PMC2224572

[r52] Pileri, P., Uematsu, Y., Campagnoli, S., Galli, G., Falugi, F., Petracca, R., Weiner, A. J., Houghton, M., Rosa, D. & other authors (1998). Binding of hepatitis C virus to CD81. Science 282, 938–941. 9794763 10.1126/science.282.5390.938

[r53] Ploss, A., Evans, M. J., Gaysinskaya, V. A., Panis, M., You, H., de Jong, Y. P. & Rice, C. M. (2009). Human occludin is a hepatitis C virus entry factor required for infection of mouse cells. Nature 457, 882–886. 19182773 10.1038/nature07684 PMC2762424

[r54] Roccasecca, R., Ansuini, H., Vitelli, A., Meola, A., Scarselli, E., Acali, S., Pezzanera, M., Ercole, B. B., McKeating, J. & other authors (2003). Binding of the hepatitis C virus E2 glycoprotein to CD81 is strain specific and is modulated by a complex interplay between hypervariable regions 1 and 2. J Virol 77, 1856–1867. 12525620 10.1128/JVI.77.3.1856-1867.2003 PMC140892

[r55] Rosa, D., Campagnoli, S., Moretto, C., Guenzi, E., Cousens, L., Chin, M., Dong, C., Weiner, A. J., Lau, J. Y. & other authors (1996). A quantitative test to estimate neutralizing antibodies to the hepatitis C virus: cytofluorimetric assessment of envelope glycoprotein 2 binding to target cells. Proc Natl Acad Sci U S A 93, 1759–1763. 8700831 10.1073/pnas.93.5.1759 PMC39854

[r56] Scarselli, E., Ansuini, H., Cerino, R., Roccasecca, R. M., Acali, S., Filocamo, G., Traboni, C., Nicosia, A., Cortese, R. & Vitelli, A. (2002). The human scavenger receptor class B type I is a novel candidate receptor for the hepatitis C virus. EMBO J 21, 5017–5025. 12356718 10.1093/emboj/cdf529 PMC129051

[r57] Schofield, D. J., Bartosch, B., Shimizu, Y. K., Allander, T., Alter, H. J., Emerson, S. U., Cosset, F. L. & Purcell, R. H. (2005). Human monoclonal antibodies that react with the E2 glycoprotein of hepatitis C virus and possess neutralizing activity. Hepatology 42, 1055–1062. 16250048 10.1002/hep.20906

[r58] Shimizu, Y. K., Hijikata, M., Iwamoto, A., Alter, H. J., Purcell, R. H. & Yoshikura, H. (1994). Neutralizing antibodies against hepatitis C virus and the emergence of neutralization escape mutant viruses. J Virol 68, 1494–1500. 8107212 10.1128/jvi.68.3.1494-1500.1994 PMC236605

[r59] Simmonds, P. (1995). Variability of hepatitis C virus. Hepatology 21, 570–583. 7531173 10.1002/hep.1840210243 PMC7165699

[r60] Tarr, A. W., Owsianka, A. M., Timms, J. M., McClure, C. P., Brown, R. J., Hickling, T. P., Pietschmann, T., Bartenschlager, R., Patel, A. H. & Ball, J. K. (2006). Characterization of the hepatitis C virus E2 epitope defined by the broadly neutralizing monoclonal antibody AP33. Hepatology 43, 592–601. 16496330 10.1002/hep.21088

[r61] Troesch, M., Meunier, I., Lapierre, P., Lapointe, N., Alvarez, F., Boucher, M. & Soudeyns, H. (2006). Study of a novel hypervariable region in hepatitis C virus (HCV) E2 envelope glycoprotein. Virology 352, 357–367. 16781757 10.1016/j.virol.2006.05.015

[r62] Vieyres, G., Angus, A. G., Haberstroh, A., Baumert, T. F., Dubuisson, J. & Patel, A. H. (2009). Rapid synchronization of hepatitis C virus infection by magnetic adsorption. J Virol Methods 157, 69–79. 19100780 10.1016/j.jviromet.2008.11.015

[r63] Voisset, C., Callens, N., Blanchard, E., Op De Beeck, A., Dubuisson, J. & Vu-Dac, N. (2005). High density lipoproteins facilitate hepatitis C virus entry through the scavenger receptor class B type I. J Biol Chem 280, 7793–7799. 15632171 10.1074/jbc.M411600200

[r64] Wakita, T., Pietschmann, T., Kato, T., Date, T., Miyamoto, M., Zhao, Z., Murthy, K., Habermann, A., Krausslich, H. G. & other authors (2005). Production of infectious hepatitis C virus in tissue culture from a cloned viral genome. Nat Med 11, 791–796. 15951748 10.1038/nm1268 PMC2918402

[r65] Weiner, A. J., Brauer, M. J., Rosenblatt, J., Richman, K. H., Tung, J., Crawford, K., Bonino, F., Saracco, G., Choo, Q. L. & other authors (1991). Variable and hypervariable domains are found in the regions of HCV corresponding to the flavivirus envelope and NS1 proteins and the pestivirus envelope glycoproteins. Virology 180, 842–848. 1846505 10.1016/0042-6822(91)90104-j

[r66] Weiner, A. J., Geysen, H. M., Christopherson, C., Hall, J. E., Mason, T. J., Saracco, G., Bonino, F., Crawford, K., Marion, C. D. & other authors (1992). Evidence for immune selection of hepatitis C virus (HCV) putative envelope glycoprotein variants: potential role in chronic HCV infections. Proc Natl Acad Sci U S A 89, 3468–3472. 1314389 10.1073/pnas.89.8.3468 PMC48889

[r67] Yanagi, M., Purcell, R. H., Emerson, S. U. & Bukh, J. (1997). Transcripts from a single full-length cDNA clone of hepatitis C virus are infectious when directly transfected into the liver of a chimpanzee. Proc Natl Acad Sci U S A 94, 8738–8743. 9238047 10.1073/pnas.94.16.8738 PMC23104

[r68] Zeisel, M. B., Koutsoudakis, G., Schnober, E. K., Haberstroh, A., Blum, H. E., Cosset, F. L., Wakita, T., Jaeck, D., Doffoel, M. & other authors (2007). Scavenger receptor class B type I is a key host factor for hepatitis C virus infection required for an entry step closely linked to CD81. Hepatology 46, 1722–1731. 18000990 10.1002/hep.21994

[r69] Zheng, A., Yuan, F., Li, Y., Zhu, F., Hou, P., Li, J., Song, X., Ding, M. & Deng, H. (2007). Claudin-6 and claudin-9 function as additional coreceptors for hepatitis C virus. J Virol 81, 12465–12471. 17804490 10.1128/JVI.01457-07 PMC2169001

[r70] Zhong, J., Gastaminza, P., Cheng, G., Kapadia, S., Kato, T., Burton, D. R., Wieland, S. F., Uprichard, S. L., Wakita, T. & Chisari, F. V. (2005). Robust hepatitis C virus infection in vitro. Proc Natl Acad Sci U S A 102, 9294–9299. 15939869 10.1073/pnas.0503596102 PMC1166622

[r71] Zibert, A., Schreier, E. & Roggendorf, M. (1995). Antibodies in human sera specific to hypervariable region 1 of hepatitis C virus can block viral attachment. Virology 208, 653–661. 7538251 10.1006/viro.1995.1196

