# Supplementary material for: Characterization of antibody-mediated neutralization directed against the hypervariable region 1 of hepatitis C virus E2 glycoprotein
Source: J Gen Virol. 2011 Mar;92(Pt 3):494–506. doi: 10.1099/vir.0.028092-0 (PMC3081231; doi:10.1099/vir.0.028092-0)
Supplement: [Supplementary Table] [file supp_92_3_494__index.html]

 Characterization of antibody-mediated neutralization directed against the hypervariable region 1 of hepatitis C virus E2 glycoprotein -- Vieyres et al. 92 (3): 494 Data Supplement - Supplementary Table -- Journal of General Virology

## 

### Characterization of antibody-mediated neutralization directed against the hypervariable region 1 of hepatitis C virus E2 glycoprotein, by G. Vieyres, J. Dubuisson and A. H. Patel

*Journal of General Virology* vol. **92**, part 3, pp. 494 - 506

**Supplementary Table S1.** Anti-HVR1 antibodies neutralize chimeric HCVpp infection **[PDF]  (50 KB)**

  
  
