# Supplementary material for: Characterization of antibody-mediated neutralization directed against the hypervariable region 1 of hepatitis C virus E2 glycoprotein
Source: J Gen Virol. 2011 Mar;92(Pt 3):494–506. doi: 10.1099/vir.0.028092-0 (PMC3081231; doi:10.1099/vir.0.028092-0)
Supplement: [Supplementary Table] [file supp_92_3_494__1.pdf]

Supplementary Table S1. Anti-HVR1 antibodies neutralize chimeric HCVpp infection

HCVpp harbouring WT H77c E1E2 or H77c E1E2 with the Gla, HG or GH HVR1 (see main text) were incubated with a range of concentrations of anti-HVR1 antibodies and the IC_{50} values ($\mu g\ ml^{-1}$) were determined as shown.

**Antibody**	**HCVpp**			
	**H77c**	**H77c-GlaHVR1**	**HG**	**GH**
AP123	>30	1.5	9	>30
R1020	1.5	7	15	2.5
R140	15	1	2	20

Vieyres, G., Dubuisson, J. and Patel, A. H. (2011). Characterization of antibody-mediated neutralization directed against the hypervariable region 1 of hepatitis C virus E2 glycoprotein. *J Gen Virol* **92**, 494–506.
